# Ultrasound-guided phlebotomy in primary care for people who inject drugs

**DOI:** 10.1186/s12954-023-00762-5

**Published:** 2023-03-23

**Authors:** Michael Huyck, Stockton Mayer, Sarah Messmer, Charles Yingling, Shirley Stephenson

**Affiliations:** 1 University of Illinois at Chicago (UIC) School of Nursing, Chicago, USA; 2 Department of Infectious Disease, UIC College of Medicine, Chicago, USA; 3 Department of Pediatrics, UIC College of Medicine, Chicago, USA; 4 University of Michigan School of Nursing, Ann Arbor, USA; 5 UIC College of Nursing, Chicago, USA

**Keywords:** Persons who inject drugs; PWID, Buprenorphine, Medication for opioid use disorder, MOUD, Hepatitis C, Venous access, Phlebotomy, Ultrasound, Opioid use disorder

## Abstract

**Objectives:**

Persons who inject drugs (PWID) commonly experience venous degradation as a complication of prolonged injection, which makes routine phlebotomy difficult. Clients may decline care due to the perceived lack of skilled phlebotomy services, and this contributes to significant delays in infectious disease screening and treatment. In this study, we investigated ultrasound-guided phlebotomy in clients with difficult venous access receiving care at two low-threshold buprenorphine clinics. Our objectives were to increase the accuracy of vascular access, expedite infectious disease treatment for hepatitis C virus (HCV) and human immunodeficiency virus (HIV), and increase client satisfaction with phlebotomy services.

**Methods:**

PWID who declined routine phlebotomy at two clinic sites were offered ultrasound-guided vascular access by a trained clinician. Participants completed a survey to collect data regarding acceptability of the intervention.

**Results:**

Throughout a 14-month period, 17 participants were enrolled. Of the total 30 procedures, 41.2% of clients returned for more than one phlebotomy visit, and 88.2% of clients achieved vascular access within 1 attempt. Of participating clients, 52.9% described themselves as having difficult venous access and at conclusion of the study, 58.8% expressed more willingness to have phlebotomy performed with an ultrasound device.

**Conclusions:**

Offering ultrasound-guided phlebotomy for PWID with difficult venous access resulted in decreased access attempts, increased patient satisfaction, and expedited screening and treatment for HIV and HCV point-of-care ultrasound technology is an effective approach to improving care for persons who inject drugs.

## Introduction

Persons who inject drugs (PWID) commonly experience venous degradation as a complication of prolonged injection [1]. Venous sclerosis and injection site vein loss are common within this population and contribute to difficult vascular access in clinical settings [2]. Intravenous drug use has been identified as a significant risk factor for difficult vascular access and it contributes to significant delays in care compared with non-injecting persons [3]. Multiple factors contribute to difficult venous access in persons injecting drugs such as repeated vessel injury, drug adulterants such as xylazine, hygienic practices, and heavily acidic formulations of heroin which may contribute to thrombophlebitis, sclerosis, and subsequent vein loss [4]. While other medical comorbidities such as obesity, diabetes, end-stage renal disease, and hypotension can necessitate the need for advance intravenous access techniques such as the use of vascular access ultrasound, intravenous drug injection is often a leading risk factor for difficult venous access [5].

The objectives of this study were to increase accuracy and decrease attempts to obtain venous access by utilizing ultrasound-guided phlebotomy in PWID who declined routine office-based phlebotomy within two low-threshold buprenorphine clinics. Laboratory studies such as human immunodeficiency virus (HIV) and hepatitis C virus (HCV) viral loads and genotyping were obtained to expedite treatment for these conditions in addition to routine laboratory work such as metabolic profiles required for treatment monitoring.

Decreased venous access among PWID is a significant risk factor in forgoing healthcare services. PWID presenting for healthcare services within the community setting have reported fear of phlebotomy failure and subsequent painful attempts as reasons for forgoing care, including HCV and/or HIV treatment [5]. The impetus for this study was a need identified by clinicians at two primary care clinics offering low-threshold buprenorphine treatment. These clinicians reported challenges in obtaining vascular access among clients who currently inject and those who previously injected drugs. Clients in the two study locations often declined routine laboratory tests and infectious disease screening due to venous access issues. Delays in care occurred when clients were unable to initiate HCV/HIV workups or missed follow-up laboratory tests during treatment.

The use of ultrasound guidance for obtaining vascular access is a well-documented technique shown to decrease time and access attempts while increasing patient satisfaction [6]. While current research mainly encompasses the use of ultrasound for the placement of intravenous catheters, the same technology can be utilized for phlebotomy. Additionally, point-of-care (POC) ultrasound technology has become more affordable and available in recent years including wider adoption in the primary car setting [7]. Such devices are capable of being operated on mobile phones and tablet devices and can easily be utilized between practice sites. Increasing the accuracy of phlebotomy services may improve the clinician-client relationship and increase patient comfort. Clients may experience less fear and apprehension knowing an experienced clinician is capable of quickly and accurately obtaining vascular access; ultimately this may improve treatment adherence.

## Methods

Individuals who self-reported currently or previously injecting drugs were invited to participate in the study. All participants came from two primary care sites located in Chicago which also provide low-threshold buprenorphine treatment. Eligible participants were individuals who declined phlebotomy due to perceived difficult access or prior experiences that required multiple access attempts with standard phlebotomy. These individuals were offered the opportunity to enroll in the study to obtain needed laboratory studies as part of ongoing care for medication for opioid use disorder (MOUD), infectious disease screening and treatment, and primary care. None of the participants who were informed of the study declined to participate. Study participants were given longer appointments designated for a procedure so that there would be adequate time to address the questions and review the informed consent. All procedures were performed with routine phlebotomy catheters and use of an ultrasound probe with tablet device. This study was reviewed by and approved by our institutional review board.

A clinician trained in ultrasound-guided vascular access utilized a portable diagnostic ultrasound device to perform phlebotomy with a standard 23-gauge 0.75-inch butterfly catheter. Vascular ultrasound training was provided to study personnel as part of an emergency department program for ultrasound-guided intravenous catheter insertion. This consisted of a one-hour class of instruction with an ultrasound device including use of a prosthetic arm with deep vessels. The technique used to identify venous structures for cannulation was then applied to routine phlebotomy in the outpatient setting. The ultrasound operator performed 20 vascular access attempts over a 1-month period to become proficient. This study utilized an FDA approved device with a frequency range 1–10 MHz to visualize vascular structures such as the superficial and deep veins of the upper extremities. The visualization of deep venous structures can facilitate selection of quality veins not superficially available in clients with difficult-to-access vasculature. Blood draws were limited to the basilic, brachial, and cephalic veins proximal and distal to the antecubital fossa**.**

After phlebotomy, participants were then offered a non-compensated ten-question Likert style survey to assess perspectives of the intervention. There was no monetary incentive for participating in the study or for completing the survey. The survey questions were developed based on prior clinical experience with difficult venous access clients within the study locations. Statements previously mentioned by clients such as: “You will not be able to get blood without an ultrasound machine or a central line; It can take 20 attempts to get blood; You’ll only be able to get blood from an artery,” were used to develop the survey questions. The Likert-based survey was utilized to assign quantitative value to the qualitative data collected regarding the patient experience. Surveys were delivered to participants post-procedure by the provider utilizing a paper form. Demographic information such as age was collected from the electronic medical record. Our team then performed a descriptive analysis of collected data.

## Results

From April 2021–June 2022, 17 participants were enrolled resulting in 30 procedures (Table 1). Combined HCV and opioid use disorder (OUD) (82.4%) were the most common diagnoses in those receiving ultrasound-guided phlebotomy services. Other conditions present among clients included HIV, alcohol use disorder (AUD), and a combination of these conditions. Some condition-specific data were excluded due to small sample size reporting restrictions. Race and ethnicity data collection were not study requirements and therefore not included in this paper. The brachial vein was the most common vessel of access (73.3%) and the most common complication was brachial arterial puncture (16.7%). Many of the clients selected for the study (41.2%) were familiar with the use of ultrasound technology for intravenous access through previous hospital admissions or emergency department visits where access was achieved with midlines and central lines. Half of clients (52.9%) reported themselves as having difficult venous access, (58.8%) expressed more willingness to have phlebotomy performed with an ultrasound device, and 41.2% returned for more than one phlebotomy visit (Table 2). Office visits for ultrasound-guided phlebotomy took an average of 30 min to complete. Vascular access was achieved within 100% of participants utilizing the ultrasound device.

**Table 1:** Demographics of Ultrasound Phlebotomy Clients: April 2021–June 2022

Wound client demographics	Total
Age	
30–79	17 (100%)
Medical indication	
HCV/OUD	14 (82.4%)
Vascular access vessel	
Brachial vein	22 (73.3%)
Basilic vein	2 (6.7%)
Median cubital vein	1 (3.3%)
Brachial artery	5 (16.7%)
Repeat phlebotomy visit	7 (41.2%)

Total Clinic Patients Seen (*N* = 17) Total phlebotomy procedures (*n* = 30)

*HCV* Hepatitis C, *HIV* Human immunodeficiency virus

**Table 2:** Survey Results of Ultrasound Phlebotomy Clients April 2021–June 2022

Statements	Strongly agree	Agree	Neutral	Disagree	Strongly Disagree	Blank
I consider myself to be a “hard stick.” My veins are generally hard to find for most healthcare personnel	9 (52.9%)	1 (5.9%)	1 (5.9%)	1 (5.9%)	4 (23.5%)	1 (5.9%)
When needing to have blood drawn it usually takes multiple attempts to obtain a sample	11 (64.7%)	1 (5.9%)	–	–	4 (23.5%)	1 (5.9%)
I have had an arterial blood draw (blood drawn from the wrist) because my veins are hard to find	7 (41.2%)	–	–	–	9 (52.9%)	1 (5.9%)
I have declined having blood drawn because it is often too painful and uncomfortable	5 (29.4%)	1 (5.9%)	1 (5.9%)	2 (11.8%)	7 (41.2%)	1 (5.9%)
I have forgone medical care and procedures because it was too difficult to obtain the required blood work	4 (23.5%)	2 (11.8%)	1 (5.9%)	1 (5.9%)	8 (47.1%)	1 (5.9%)
When being admitted to the emergency room or hospital an ultrasound device has been used on me to place an IV or draw blood	7 (41.2%)	3 (17.1%)	–	–	6 (35.3%)	1 (5.9%)
I am comfortable with healthcare providers using an ultrasound device to draw blood or place IV’s	11 (64.7%)	–	–	–	5 (29.4%)	1 (5.9%)
It would be acceptable to use an ultrasound device to draw blood in my primary care provider’s office	11 (52.9%)	–	–	–	4 (23.5%)	2 (11.8%)
I would be more willing to have blood drawn if I knew an ultrasound device was available	10 (58.8%)	1 (5.9%)	–	–	5 (29.4%)	1 (5.9%)
I would be willing to book an appointment to have my blood drawn with an ultrasound device by a trained healthcare provider	10 (58.8%)	1 (5.9%)	–	–	5 (29.4%)	1 (5.9%)

*IV* Intravenous line

## Discussion

Difficulty achieving vascular access is a common complication in current and former PWID which has been successfully addressed with the use of vascular ultrasound mainly in the Emergency Department setting [3, 7]. Ultrasound-guided phlebotomy can be readily instituted with point-of-care ultrasound in the outpatient setting as evidenced in this study. The technique assures high accuracy, patient satisfaction, and return visits. Anxiety, embarrassment, and stigmatization were common themes among clients describing prior experience with phlebotomy staff. Multiple painful access attempts by healthcare staff, blind draws, and shaming were also mentioned as contributing factors to the avoidance of infectious disease treatment initiation, follow-up, and routine primary care.

Phlebotomy represents a significant barrier to accessing routine care for persons who inject drugs [6]. A clinic-level vascular access program requires the evaluation and selection of clinical personnel with advanced vascular access skills including the use of ultrasound. While accounting for slightly more clinical time to perform the procedure, it is billable under current procedural terminology (CPT) and can be utilized as a value-added service. The brachial vein was the largest and most superficial vessel present and accounted for the majority of obtained specimens. Brachial vein access also contributed to most inadvertent brachial arterial punctures as the vessels flank one another in proximity. In the cases of arterial puncture, phlebotomy was still performed and there were no major bleeding complications among participants. The likelihood of this complication can be decreased with newer probes which contain biplane imaging. The probe utilized for this study was a multisystem device which can also affect accuracy compared with vascular-specific probes. The use of radiopaque catheters can also further increase accuracy with the drawback of increased cost. Arterial puncture is not common in routine phlebotomy where only superficial vessels are accessed. All access within this study was achieved within a subcutaneous depth of 0.5–1.5 cm.

Limitations of this study include generalizability as procedures were performed at both practice sites by a single clinician. Procedural accuracy may be variable across providers and level of training. Barriers expressed by participants may not be applicable to all PWID. There is also a potential for bias as in most cases the ultrasound operator was also the treating clinician and had developed rapport with patients. Literacy level may have contributed to effects in survey data collection as the collection instrument was offered on paper in English only. All enrolled participants spoke English but reading and comprehension was not assessed.

## Conclusion

This study demonstrates that readily available and affordable POC ultrasound technology can be used in the office-based setting to achieve targeted venous access in special populations such as PWID. Vascular access can also be achieved with the use of standard phlebotomy equipment. Most study participants voiced satisfaction with the procedure and indicated willingness to follow up for other medical needs. Offering ultrasound-guided phlebotomy for PWID with difficult venous access resulted in decreased access attempts, increased patient satisfaction, and expedited screening and treatment for HIV and HCV. Point-of-care ultrasound technology is an effective approach to improving care within the PWID population.

## Data Availability

The datasets generated and/or analyzed during the current study are not publicly available due to the protection of the participants’ privacy but are available from the corresponding author on reasonable request.

## Abbreviations

AUD: Alcohol use disorder
CPT: Current procedural terminology
HCV: Hepatitis C
HIV: Human immunodeficiency virus
MOUD: Medication for opioid use disorder
OUD: Opioid use disorder
PWID: Persons who inject drugs
POC: Point of care
